# A 63‐kg giant neurofibroma in the right lower extremity and gluteal region of a 22‐year‐old woman: A case report

**DOI:** 10.1002/ccr3.4152

**Published:** 2021-05-04

**Authors:** Samit Sharma, Biraj Pokhrel, Namrata Khadka, Sangam Rayamajhi, Jayan Man Shrestha, Ishwar Lohani

**Affiliations:** ^1^ Department of Plastic Surgery Tribhuvan University Institute of Medicine Kathmandu Nepal

**Keywords:** ancillary procedures, giant neurofibroma, intraoperative hemorrhage, lower extremity, staging of the surgery

## Abstract

Excessive intraoperative hemorrhage in the management of a giant neurofibroma can be reduced with ancillary procedures such as ligation of the feeding/nutrient artery, adopting proper intraoperative hemostatic methods, and by staging the surgery.

## Graphical abstract

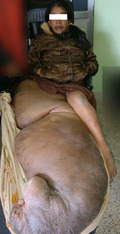

## 1 INTRODUCTION

A 22‐year‐old woman presented with progressive and massive swelling of her right lower extremity and gluteal region since birth and was rendered nonambulatory for the past 4 years by the tumor. She underwent resection of 63 kgs of the tumor in two stages, without significant intraoperative blood loss.

A neurofibroma is a benign nerve‐sheath tumor especially occurring in the peripheral nervous system. However, these tumors can grow anywhere in body, including the skin, soft tissue, visceral organs, brain, and spinal cord. They arise from the cells and tissues that cover the nerves. Neurofibromas can be divided into (a) Solitary or isolated neurofibromas, which originate from cutaneous nerves; (b) Diffuse, arising from the nerves in the subcutaneous tissues; (c) plexiform, which are diffuse masses with tortuous expansion along the branches of the parent nerve.^1^, ^2^

Although most of the neurofibromas are sporadic, some people may have a genetic condition known as neurofibromatosis (NF). Up to 10% of the neurofibromas are associated with NF.^3^ Neurofibromatosis is a group of genetic disorders inherited as an autosomal dominant trait. NF can be classified into three types, including NF1, NF2, and schwannomatosis.^4^, ^5^ NF1, also known as Von Reklinghausen's disease or peripheral neurofibromatosis, always involves skin and soft tissue and affects nearly 1/4950 individuals.^6^, ^7^ Although a genetic disorder, 50% of cases arise from spontaneous mutations with no family history.^8^ NF1 is linked to a large gene on band *17q11.2*. It encodes a protein called neurofibromin, which may act as a tumor suppressor.^9^ There is a two to five percent chance of malignant transformation of NF1 to Malignant Peripheral Nerve Sheath Tumors (MPNST).^10^ Two of the following clinical features are required for the diagnosis^11^:

- Six or more cafe au lait macules (>0.5 cm in children and >1.5 cm in adults
- Two or more cutaneous/subcutaneous neurofibromas or one plexiform neurofibroma
- Axillary or groin freckling
- Optic pathway glioma
- 2 or more Lisch nodules (iris hamartomas seen on slit‐lamp examination)
- Bony dysplasia (sphenoid wing dysplasia, bowing of long bone with or without pseudoarthrosis)
- First degree relative with NF1

Giant neurofibroma is the term used to describe the neurofibroma which has grown to a significant size. Authors use this term to refer to those neurofibromas when the neurofibroma weighs 20% or more of the patient's bodyweight.^12^ Herein, we report a case of a massive, giant neurofibroma affecting the right lower extremity of the patient, who underwent above knee amputation in the first stage and debulking of the tumor in the second stage.

## 2 CASE PRESENTATION

**FIGURE 1:**
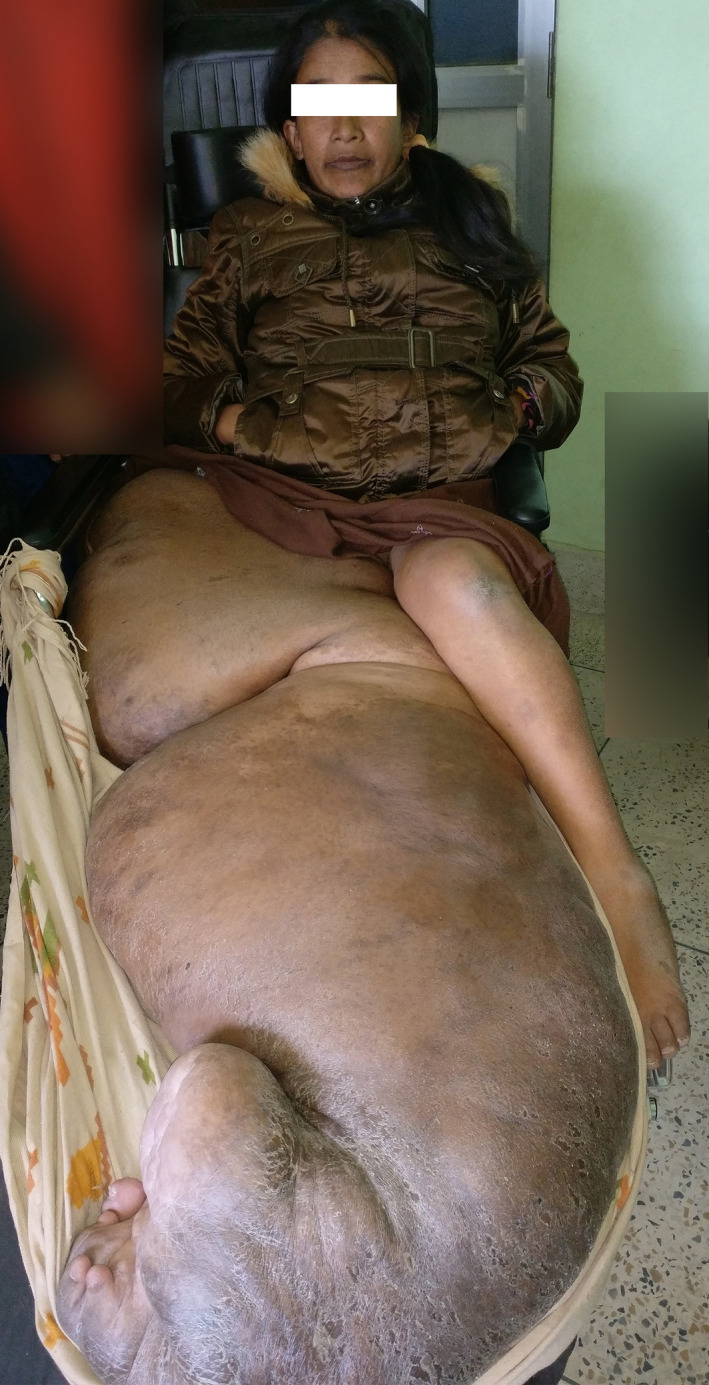
The patient with the tumor in custom‐made chair when she presented in the Out‐patient department

A 22‐year‐old woman from the remote Dolakha district of Nepal presented with a gigantic swelling of her right lower limb and gluteal region in the outpatient department of the Department of Plastic Surgery, Tribhuvan University Teaching Hospital, Kathmandu, Nepal. The mass was present since birth and had progressively increased in size, more so in the past 4 years making it impossible for her to walk (Figure 1). There was no family history of neurofibromatosis. Though she had problems in ambulation since early childhood and used to walk with a limp, it was only after she could not walk that she sought for medical help. The delay in seeking medical help was also attributable to the very poor socioeconomic condition of the patient. The patient had to be transferred in a large custom‐made wheelchair and needed the assistance of three to four people for any form of transfer.

The patient noticed multiple hyperpigmented lesions and multiple painless nodular lesions in her body since childhood. She dropped out of school when she was studying in grade six, not only due to the disability of having to walk a long distance to reach her school but also because she was stigmatized in the society. She suffered from peer teasing and had no friends. The people in her village thought that she had contagious elephantiasis and that by playing with her, their children would contract the disease.

**FIGURE 2:**
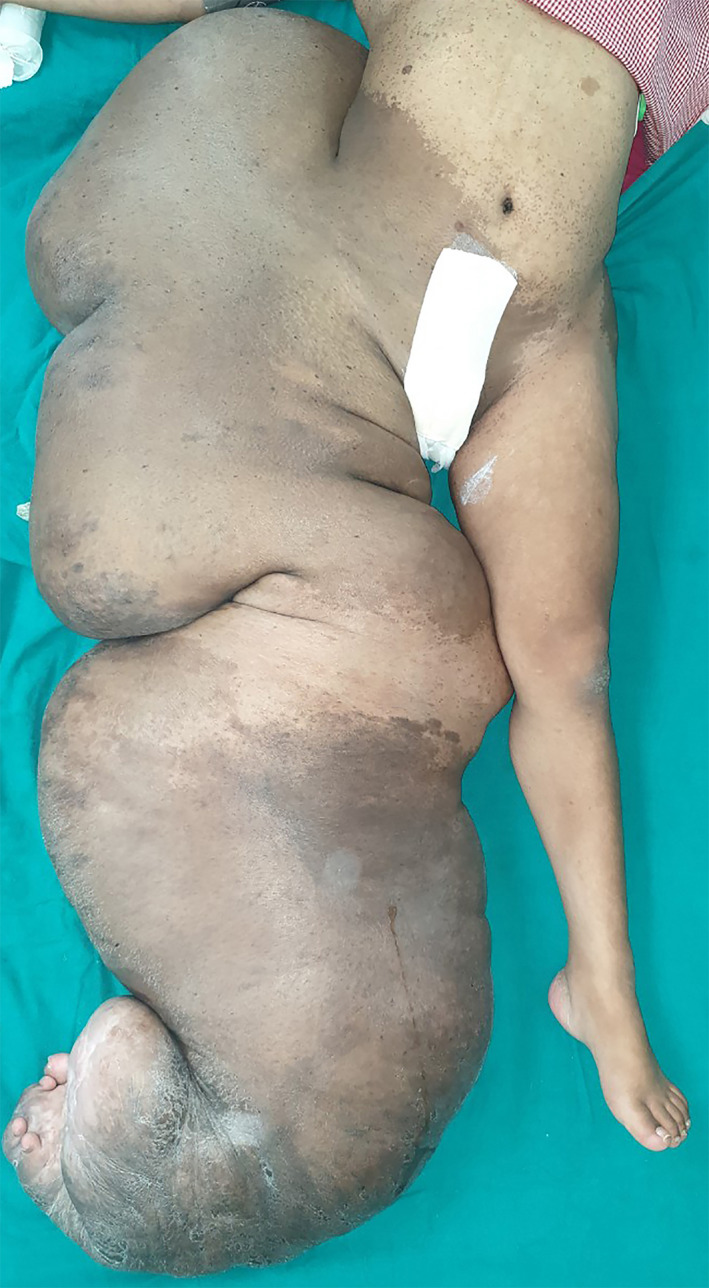
The patient on the operating table

Physical examination revealed a large, firm, soft tissue mass measuring 145 cms in length and 40 cms in width involving the entirety of the right lower limb, gluteal region, waist, and perineal region (Figure 2). The skin overlying the gluteal region and the calf had giant Cafe‐au‐lait spots. The hip, knee, and ankle joints were dislocated, and the limb was in externally rotated position. The patient showed appreciable movements only in the toes. The largest circumference of the right thigh was 72 cms and that of the right leg was 76 cms.

**FIGURE 3:**
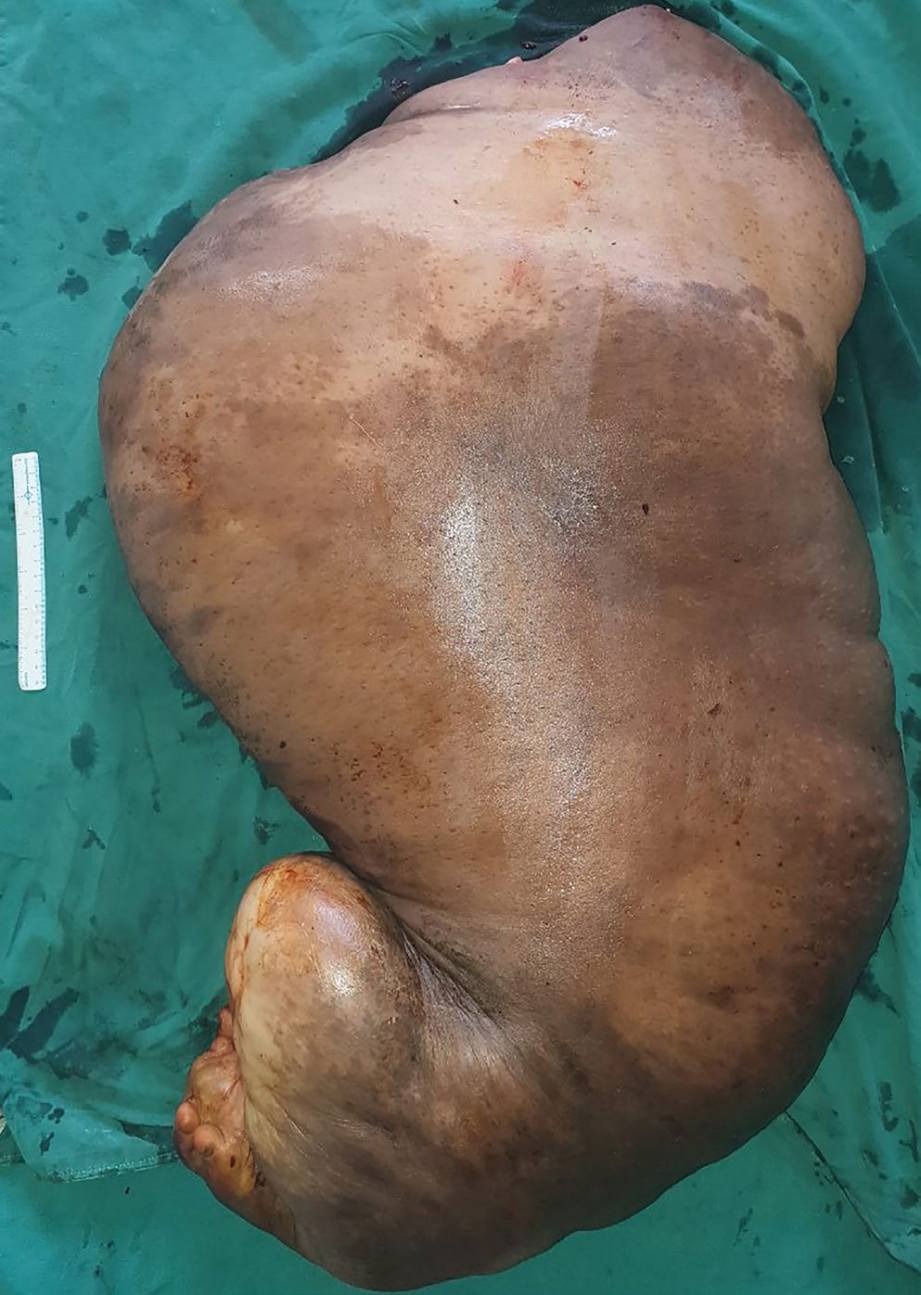
The amputated specimen following above knee amputation which weighed 35 kgs

**FIGURE 4:**
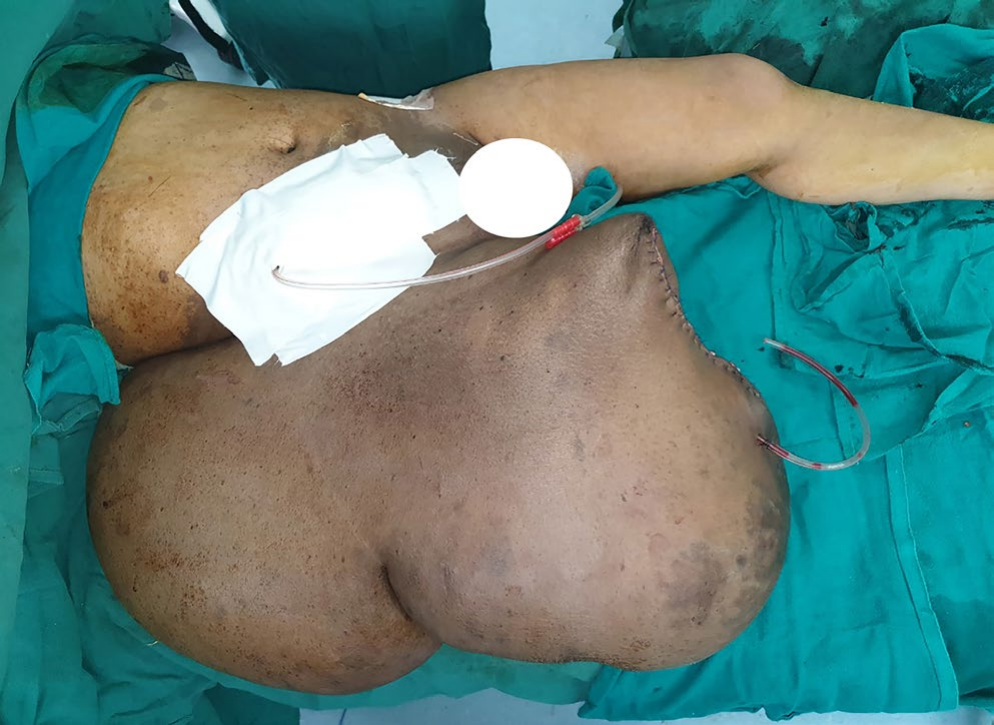
The stump closed over suction drain

The patient underwent debulking surgery in two stages due to the extra‐large size of the limb and due to the fear of massive blood loss. Her preoperative blood parameters were within normal limits. Her hemoglobin before the first surgery was 9.3 gm%. Patient underwent the first surgery (above knee amputation through the most narrowed portion of the leg) under general anesthesia (Figure 3). The area of the operating table was extended with the help of a trolley to accommodate the whole body of the patient. In supine position, through right lower abdominal incision and retroperitoneal approach, first, the right common iliac artery was ligated in continuity so as to lessen the blood loss during surgery and also as a proxy for a tourniquet as the use of normal tourniquet was not possible due to the extra‐large circumference of the thigh. After amputation, closure of the stump and the abdominal incisions were done over tube suction drains (Figure 4). The debulked/amputated specimen weighed 35 kgs.

The postoperative period was uneventful except for the reduced hemoglobin levels of 8 gm% which was managed with one unit (350 mL) of blood transfusion. The blood loss was 300 mL.

**FIGURE 5:**
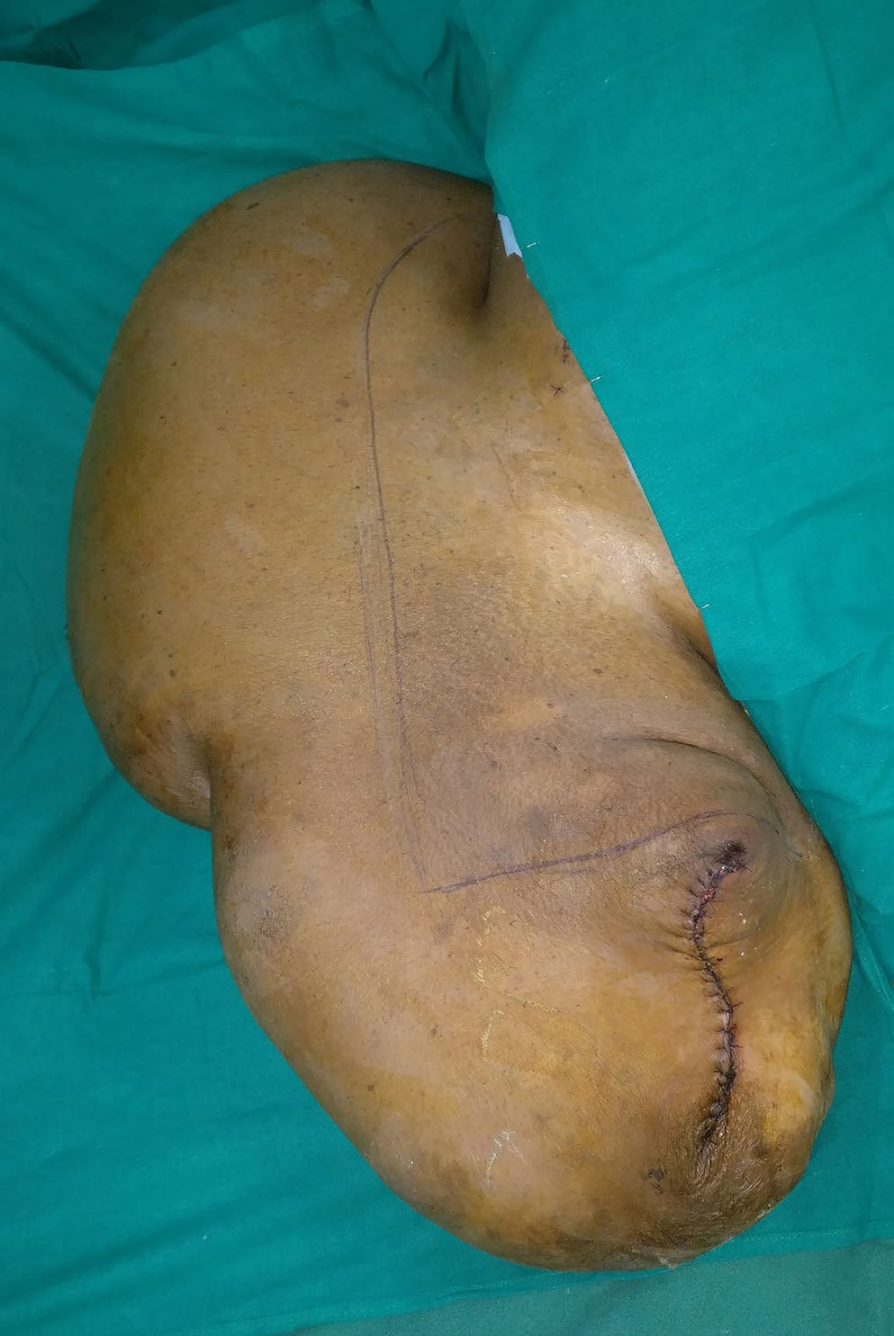
L‐shaped incision for the second stage of debulking surgery

**FIGURE 6:**
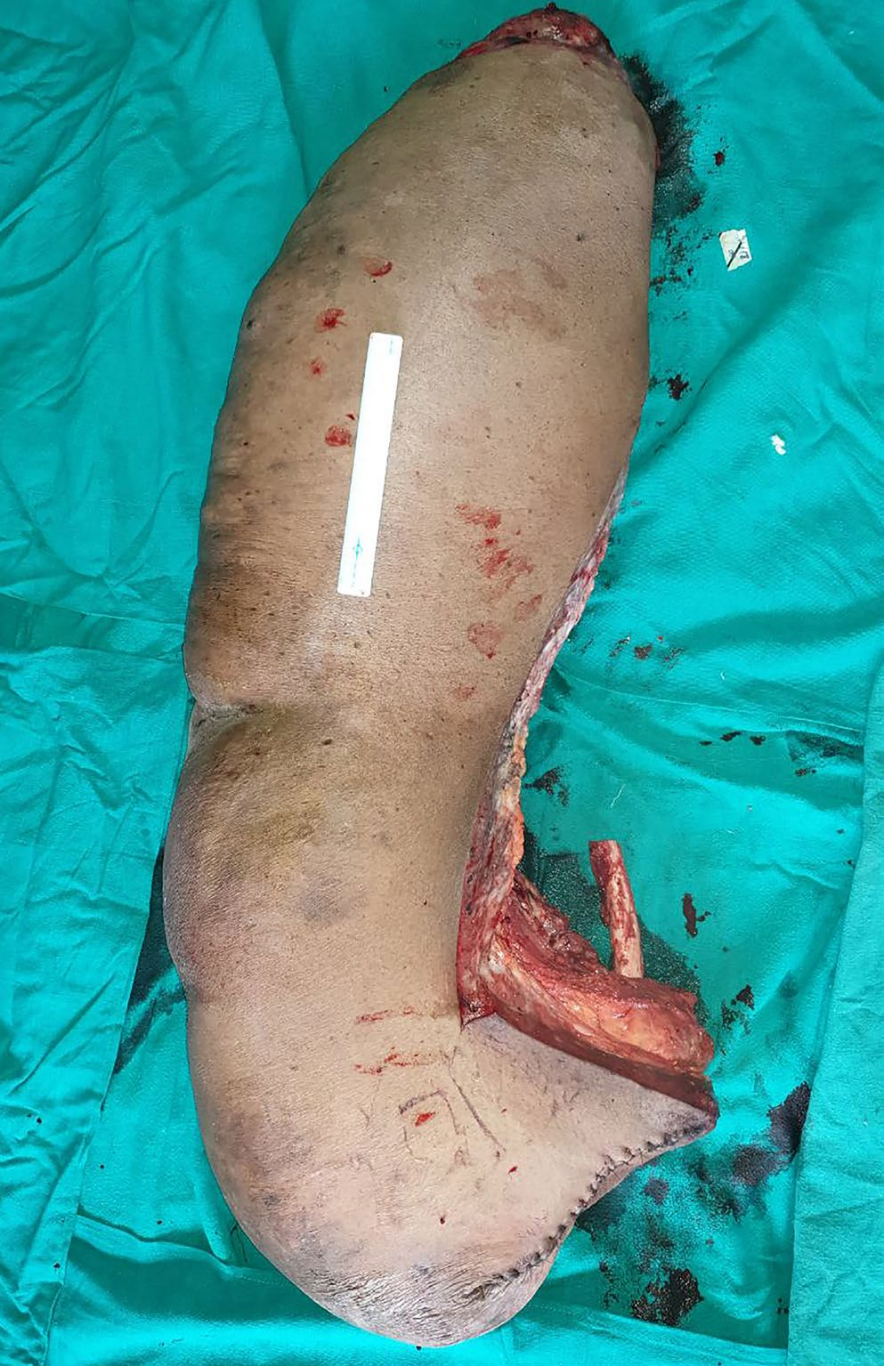
The resected specimen weighing 28 kgs after second stage surgery

**FIGURE 7:**
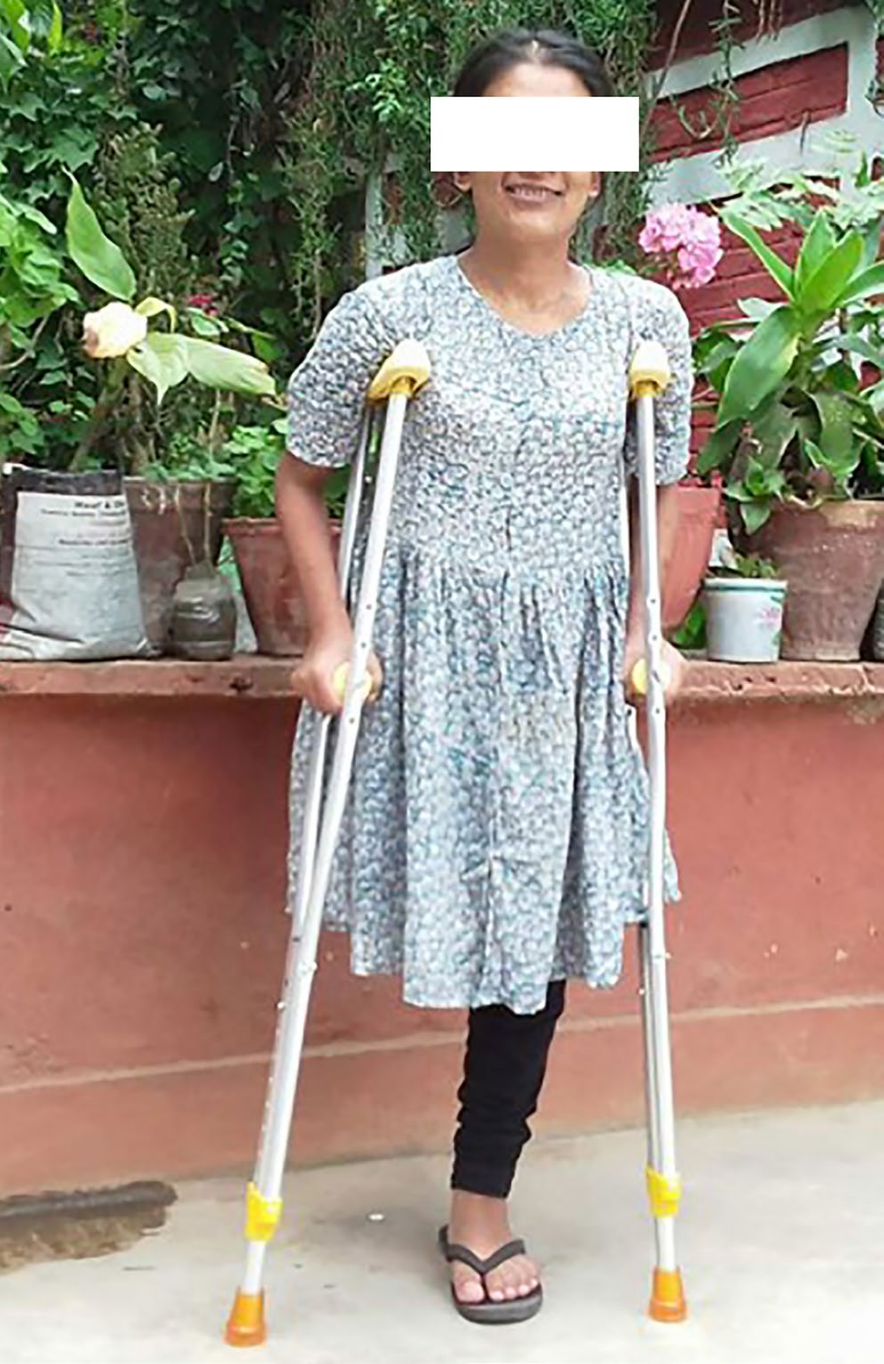
The patient ambulatory on crutches after 3 months of discharge from the hospital

The patient underwent the second stage of debulking surgery a week later. Her hemoglobin before the second surgery was 8.5 gm%. Again, under general anesthesia and in supine position, an L‐ shaped incision was given through the skin and subcutaneous tissue (Figure 5). The subdermal tissue and subfascial tissue seemed to be very vascular with dense vessels most of which were ligated. The involved tissue seemed to have a somewhat dish water‐like color. The incision proceeded through the atrophic muscles and amputation was done at the level of greater trochanter after ligation of the femoral vessels. The bony stump was covered by muscles and stump was closed mobilizing the anterior and posterior flaps over suction drains. The resected specimen weighed 28 kilograms (Figure 6). Resected tissue was sent for histopathology and the diagnosis as neurofibroma was confirmed. The postoperative period after the second surgery was also uneventful expect for the reduced hemoglobin levels of 7.2 gm%, which again was managed with two units of blood transfusion. Blood loss during the second surgery was 350 mL. The patient was able to ambulate 2 weeks after the surgery on crutches. Three months after the surgery, patient was satisfied with the outcome. (Figure 7).

## 3 DISCUSSION

When the neurofibroma continues to grow and expand and becomes giant (>20 kgs or comprising >20% of body weight), it is a big burden for patients. Early childhood, puberty, and child‐bearing age are considered to be the periods of greatest risk for disease progression. Major complications of neurofibromas include malignant differentiation and potentially life‐threatening hemorrhage whereas minor complications include local infections and wound complications.^12^ Bouts of repeated bruising and bleeding, and prolonged ulcer may distress the patients and impact their quality of life, as was seen in our case. Surgical management is still the most effective method to control or sometimes even cure this condition.^13^, ^14^ The surgery is aimed at reducing the burden due to the disease and improving function and cosmesis. The tumor tissue should be removed as much as possible and the skin tissue could be left in place. However, the challenge remains on the risk of excessive bleeding during the operation and the difficulty in repairing the huge wound after debulking.^15^ Surgical management of plexiform neurofibromas is difficult because these tumors are extensively infiltrative and highly vascularised.^12^ Complete resection is often difficult because of the extensive and infiltrative nature of these lesions, so debulking remains the mainstay of the surgical management.

Massive intraoperative hemorrhage remains the main challenge of surgical management of this disease. These excessive hemorrhages, sometimes life‐threatening, is caused by the rupture of the friable vasculature secondary to arterial dysplasia or vascular invasion by the neurofibroma.^16^, ^17^ Intraoperative hemorrhage cannot be easily controlled because the neurofibromatous tissue contains many blood sinuses with thin and poorly contractible sinus cavities.^18^ Thus ancillary procedures such as preoperative interventional embolization of tumor's nutrient arteries^12^, ^15^, ^18^ or sometimes even vessel ligation as done in our case should be considered to minimize intraoperative blood loss. Interventional embolization was not possible at our center due to the extra‐large size of the tumor and the technical difficulties in transferring and carrying out the procedure on a person with such dimensions. Blood loss was not very significant in our case and the patient was never severely anemic (Hb <7 gm%). However, large‐volume blood arrangement is necessary before the surgery of such patients.

Another important strategy adopted for minimizing blood loss during the surgery of such huge neurofibroma was carrying out the surgery in stages. In the first surgery, above knee amputation was performed and then in the next setting debulking of the thigh and gluteal region was done. A similar case has been reported in People's Republic of China, where a 26‐year‐old woman underwent resection of a giant neurofibroma in the right lower limb.^18^ The first debulking removed 21.5 kg of the tumor which was then skin grafted, but after the skin graft loss due to infection, she underwent amputation through the knee joint.

## 4 CONCLUSION

A 22‐year‐old woman underwent debulking of a 63‐kg giant neurofibroma in the right lower extremity and gluteal region (rare in its size and location) without significant blood loss mainly attributable to the ancillary measures aimed at reducing intraoperative blood loss such as ligation of feeding artery, and also attributable to performing the surgery as a staged procedure. The surgical management of such a large and giant neurofibroma reduces the tumor burden, rehabilitates the appearance and function, and aims at improving the life of the patient, when done with the help of ancillary procedures and by staging the surgery.

## CONFLICT OF INTEREST

The authors declare that there is no conflict of interest regarding the publication of this paper.

## AUTHOR CONTRIBUTIONS

SS: involved in concept, collecting information, and manuscript writing. SS, BP, and NK: participated in the literature review and edited the draft. SS, SR, JS, and IL: involved in patient care team and also independently reviewed the manuscript. SS, BP, and NK: re‐edited the draft and reshaped it into this manuscript. All authors approved the final version of the manuscript and agree to be accountable for all aspects of the work in ensuring that questions related to the accuracy or integrity of any part of the work are appropriately investigated and resolved.

## CONSENT FOR PUBLICATION

Written informed consent was obtained from the patient for publication of this case report and any accompanying images. A copy of the written consent is available for review by the editor‐in‐chief of this journal.
